# Viromes of Hungarian Peach Trees Identified by High-Throughput Sequencing of Small RNAs

**DOI:** 10.3390/plants11121591

**Published:** 2022-06-16

**Authors:** Daniel Barath, Nikoletta Jaksa-Czotter, Tunde Varga, Eva Varallyay

**Affiliations:** Genomics Research Group, Department of Plant Pathology, Institute of Plant Protection, Hungarian University of Agriculture and Life Sciences, 2100 Gödöllő, Hungary; barathdani@gmail.com (D.B.); jaksa-czotter.nikoletta@uni-mate.hu (N.J.-C.); varga.tunde07@gmail.com (T.V.)

**Keywords:** small RNA, HTS, peach, virus, diagnostics, survey, fruit tree, *Prunus* genus, PLMVd, RT-LAMP

## Abstract

Peach trees can be infected with viruses and viroids. As we do not have efficient plant protection methods against these pathogens, the prevention of infection is crucial. Fruit trees are maintained by vegetative propagation. Planting material such as certified mother trees and rootstocks should be free from viruses and viroids, and this status has to be regularly checked to prevent infections. We surveyed certified peach trees for the presence of viruses and viroids using small RNA high-throughput sequencing (HTS), an unbiased virus diagnostic method. The results of the bioinformatic analysis of HTS were validated by other molecular methods including RT-PCR, Northern blot hybridization and loop-mediated isothermal amplification (LAMP). We found the presence of plum pox virus and peach latent mosaic viroid (PLMVd) in the vector-free isolator houses, whose presence should be regularly tested. Moreover, we detected frequent infection with recently described viruses such as nectarine stem pitting-associated virus and peach-associated luteovirus (PaLV). During the survey, PLMVd and PaLV were detected for the first time in Hungary. The analysis of the presenting virus variants and possible sources of infection suggests that the source of the viral infection could be the infected propagating material. Our study emphasizes the importance of using sensitive and trustworthy diagnostic techniques to be able to detect viral infections and successfully prevent their spread by propagation material.

## 1. Introduction

Peach is a globally widespread and important temperate stone fruit tree belonging to the *Prunus* genus. Cultivars are propagated vegetatively by grafting, and are cultivated all over the world. Several viruses and viroids can infect peach, and they cannot directly be controlled by chemical applications [1]. To prevent viral spread, the international trade of plant material is rigorously controlled. Although the selection of tolerant or resistant cultivars can be an option to avoid damage caused by infection, the choice of cultivar depends on other favorable characteristics of the fruit. If cultivar selection is not an option, we still have several control strategies: the exclusion of the viruses by (i) crop quarantine and certification, (ii) the eradication of infected cultivars and rootstocks, (iii) elimination from the planting material and (iv) the control of their vectors [2]. These control measures are based on sensitive, virus-specific diagnostic techniques. For these assays, either serological enzyme-linked immunosorbent assay (ELISA) or molecular reverse transcription–polymerase chain reaction (RT-PCR) methods can be used, but they are only able to detect previously described pathogens. Being able to diagnose all of the presenting pathogens, high-throughput sequencing (HTS)-based methods have opened new possibilities for virus detection [3,4,5]. HTS can analyze the virus itself, when total nucleic acids, double-stranded RNA or nucleic acids from purified virions are sequenced. As an alternative, virus-derived small RNAs produced by the plant’s antiviral RNAi can also be determined by HTS [6]. The use of HTS for crop virus surveys revealed the presence of an unanticipated number of previously described and new viruses and generated questions about their molecular characterization and quarantine measures that remain unanswered [7,8,9].

Peach can be infected by several viruses and viroids; among them, apple chlorotic leaf spot virus (ACLSV), plum pox virus (PPV), Prunus necrotic ringspot virus (PNRSV) and nectarine stem pitting-associated virus (NSPaV) [10] have been reported in Hungary. In recent years, a number of new, peach-infecting viruses were described, mainly as a result of the use of HTS technologies [7,9].

Plum pox virus (PPV), a potyvirus that infects *Prunus* species, is one of the ten most devastating plant viruses [11]. Its infection can cause severe symptoms and the complete loss of fruit. As it is easily spread by its aphid vectors, certified nurseries have to be kept under an isolator and should be regularly checked for the presence of the virus to prevent its further spread by the propagating material [12].

Aside from PPV, peach is heavily endangered by the infection of a viroid: peach latent mosaic viroid (PLMVd). Although the infection of PLMVd is frequently latent, it is the causative agent of peach latent mosaic (PLM) disease, first described in France and later in Japan and in the USA [13]. As a member of the family *Avsunviroideae*, PLMVd has a very definite secondary structure, with a long hammerhead and eight more stem-loop arms. Frequent mutations within its genome are usually maintained only if this specific secondary structure is not, or is only slightly, altered [14]. PLMVd can reach the shoot tips, so it is easily transmitted during vegetative propagation. Certified trees should be free from PLMVd, and the viroid presence has to be regularly checked, because if infected, they are more sensitive to both biotic and abiotic stresses and their lifetime can be shortened. Viroids do not produce proteins and serological methods cannot be used for their detection. For this reason, regular tests are usually performed by biological assays on a sensitive GF305 indicator. The most straightforward visual symptom is caused by the peach calico (PC) PLMVd strain that induces a severe albinism in peach leaves. The PLMVd-PC variant contains a 12-nt insertion located at the edge of the hammerhead arm where the PC pathogenic determinant was identified. PLMVd-siRNAs derived from this region target for cleavage and degradation the host chloroplast HSP90 mRNA (cHSP90), a gene involved in chloroplast biogenesis and development, via an RNA silencing mechanism, causing the albino phenotype [15]. In addition to biological assays, other viroid-specific diagnostic methods have been reported for PLMVd. Among them, LAMP offers a very fast and sensitive alternative [16,17].

In recent years, the use of HTS revealed the presence of new viruses, such as nectarine stem pitting-associated virus (NSPaV) and peach-associated luteovirus (PaLV), which infect peach.

NSPaV was first described in nectarine cultivars imported from France to California [18] and in nectarine trees in Washington State on the East coast, originating from California [19]. The virus was present in symptomless trees at the quarantine stations tested by HTS methods (dsRNS or ribodepleted RNA seq). Since then, its presence has been described in China [20] (full genome [21]), South-Korea [22] (full genome [23]) from symptomless nectarine trees and in Japan from symptomatic *Prunus mume* trees [24]. It has been reported in Hungary in a symptomatic orchard [10]. NSPaV was found in Australia during a virus diagnostics survey sampling random *Prunus* trees [25]. Its name suggests a direct correlation with the presence of stem-pitting symptoms [18], which could not be supported in further studies. It is still not clear if the presence of stem-pitting disease can be a manifestation of the coinfection of NSPaV with nectarine virus M (NeVM) or it is a direct phenotypic expression of an answer in sensitive varieties [19].

PaLV has been described in the USA, in a quarantine station, from two imported peach varieties [26] by RNAseq. Apart from these cultivars, Konela and IVIM18, originating from Georgia and Spain, it has been described in a symptomatic nectarine orchard in Italy [27] and in symptomatic peach [28] and nectarine trees in China [21]. Although the sampled trees in these cases showed different symptoms, such as yellowing and mosaic fruit pitting, PaLV was detected in them as a coinfection with other viruses and viroids, indicating no direct correlation between its presence and the symptoms that emerged.

**Figure 1:**
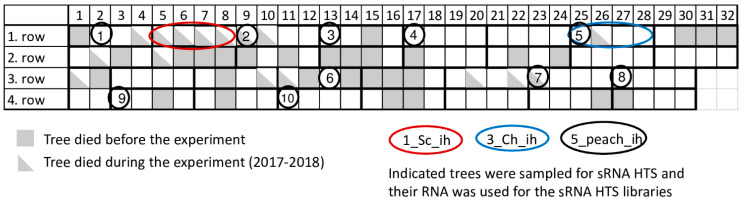
Schematic representation of the isolator, where investigated peach trees were grown.

Certified peach trees in our research station are kept in an isolator house (Figure 1) or at a nearby open field. During the past few years significant tree decline occurred in the isolator house. To find out if this decline can be connected to virus infection or not, a virus diagnostic survey was carried out by small RNA HTS, as an unbiased method. As a result, infections with several viral pathogens were identified, which were validated by RT-PCR, Northern blot hybridization or RT-LAMP assay, but which failed to be identified using a biological assay. A systematic survey of the population of certified mother trees kept in an isolator revealed a high infection rate, which could not be correlated with the observed tree decline.

## 2. Results

### 2.1. Small RNA HTS Revealed the Presence of Both Regulated and Non-Regulated Viruses

In 2017 when we started our sampling, 31 places (24%) in the isolator house were empty, indicating tree loss. To find out the reason for this decline, we sampled peach trees and carried out sRNA HTS (Figure 1 and Table 1).

The surveyed trees were not showing any virus specific symptoms. As a result of Illumina sequencing, we obtained 9.7–18.1 million raw reads (Table S1.). Their quality was checked and after trimming we used 9.65–17.7 million reads for virus diagnostics. SRNA HTS analysis showed the presence of PPV, NSPaV, PaLV, cherry-associated luteovirus (ChaLV) and PLMVd, and the results are detailed and discussed below (Table 2).

### 2.2. PPV Is Present in the Isolator

**Figure 2:**
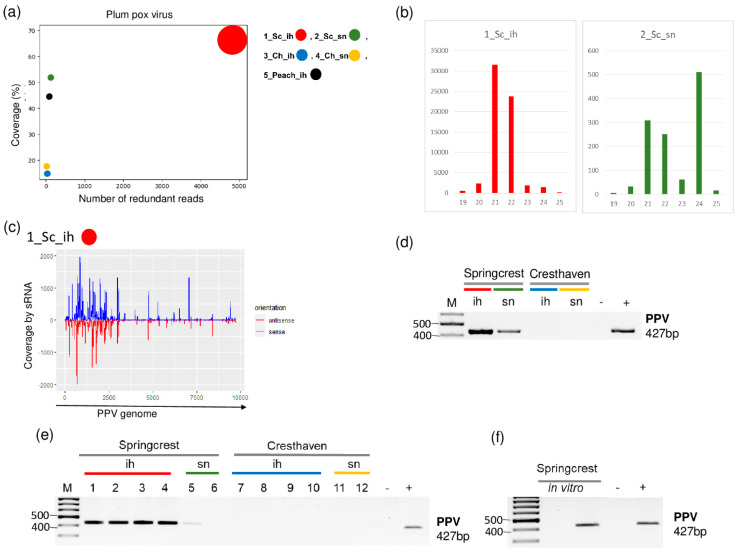
The result of the sRNA HTS and its validation for PPV in the five sRNA libraries. (**a**) The graphic representation of the result of the bioinformatics analysis of sRNA HTS. The coverage of the PPV genome (*y*-axis) together with the number of RPM (number of PPV-specific non-redundant reads in 1 million sequenced reads, on the *x*-axis) was plotted as a circle whose diameter is proportional to the number of contigs. Different colors were used for the five libraries, as indicated. (**b**) The size class distribution of the PPV-derived reads in 1_Sc_ih and 2_Sc_sn libraries. (**c**) PPV-derived reads in the 1_Sc_ih library are shown as they mapped to the PPV genome, indicating their sense and antisense origin. (**d**) The result of RT-PCR validation for the presence of PPV in the 1–4 sRNA HTS libraries using PPV-specific primers amplifying a 427 bp virus-specific amplicon. (**e**) The result of the RT-PCR validation for the presence of PPV in individual trees of 1–4 sRNA libraries using virus-specific primers amplifying a 427 bp virus-specific amplicon. (**f**) The result of the RT-PCR validation of PPV in in vitro plantlets whose ancestors were used, using PPV-specific primers amplifying a 427 bp virus-specific amplicon. M is a GeneRuler 100 bp from Thermo Scientific (Waltham, MA, USA). Sc: Springcrest; Ch: Cresthaven; ih: isolator house; sn: stock nursery, + is a positive control while − is a negative control.

Small RNA HTS in the cultivar Springcrest indicated the presence of PPV. In 1_Sc_ih, we found 94 PPV-specific contigs, and the read per million reads (RPM) and the coverage of the genome were 4908 and 68.3%, respectively. On the other hand, in 2_Sc_sn, one PPV-specific contig was detected with 122 RPM and 52% coverage of the PPV genome, respectively (Table 2 and Figure 2).

The size distribution of the PPV-derived sRNAs were slightly different in the two libraries. In 1_Sc_ih, most of the PPV-specific contigs were 21–22 nt long, while in 2_Sc_sn, beside 21–22 nt long reads, a peak at 24 nt appeared (Figure 2b). The investigation of the size distribution of small RNA reads in PPV-infected plants showed they are usually 21-22 nt long [6]. The validation of small RNA HTS was successful in both libraries. We obtained a virus-specific product with RT-PCR (see Table S2 for the sequence of the used primers) amplifying part of the viral coat protein (CP) (Figure 2d). Testing individual trees whose RNA was included in the sRNA HTS library showed that all of the individuals in 1_Sc_ih, but only one tree in 2_Sc_sn, were infected, which could explain the difference in the numbers of the PPV-derived sRNA reads between the two libraries (Figure 2e). This unusual pattern, i.e., a higher degree of infection in the isolator than in the open field, raised the question about the primary origin of the infection. Peach trees were sanitized by in vitro tissue culture; however, as they show high sensitivity to increased temperature, they were not further heat-treated. Trees in the isolator were descendants of in vitro plants, and these lines are maintained in vitro. Testing two of these in vitro lines for the presence of PPV showed that one line out of two is infected by PPV (Figure 2f). This fact suggests that in this case, the source of infection could be the in vitro cultured cultivar itself which was maintained in the isolator.

### 2.3. Luteoviruses: NSPaV and PaLV Are Present Both in the Isolator and in the Open Field

**Figure 3:**
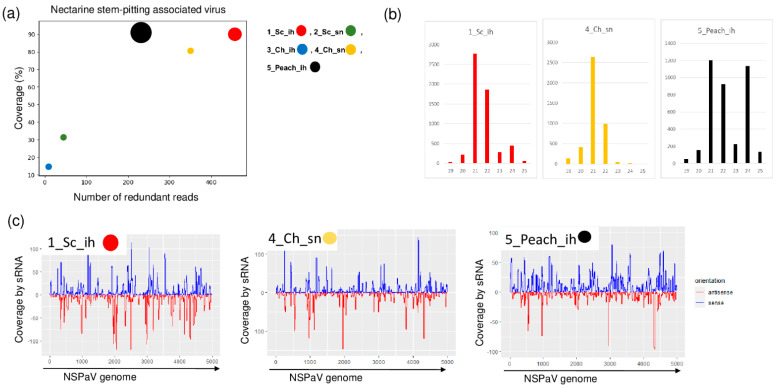
The result of the sRNA HTS and its validation for NSPaV in the five sRNA libraries. (**a**) The graphic representation of the result of the bioinformatics analysis of sRNA HTS. The coverage of the NSPaV genome (*y*-axis) together with the number of RPM (number of NSPaV-specific non-redundant reads in 1 million sequenced reads on the *x*-axis) was plotted as a circle whose diameter is proportional to the number of contigs. Different colors were used for the five libraries, as indicated. (**b**) The size class distribution of the NSPaV-derived reads in 1_Sc_ih, 4_Ch_sn and 5_Peach_ih libraries. (**c**) NSPaV-derived reads in 1_Sc_ih, 4_Ch_sn and 5_Peach_ih libraries are shown as they mapped to the NSPaV genome, indicating their sense and antisense origin (Sc: Springcrest; Ch: Cresthaven; ih: isolator house; sn: stock nursery).

NSPaV-specific contigs were detected in 1_Sc_ih and in 5_Peach_ih, but the coverage of the viral genome by sRNA reads was also higher than 80% in the case of 4_Ch_sn (Table 2 and Figure 3).

**Figure 4:**
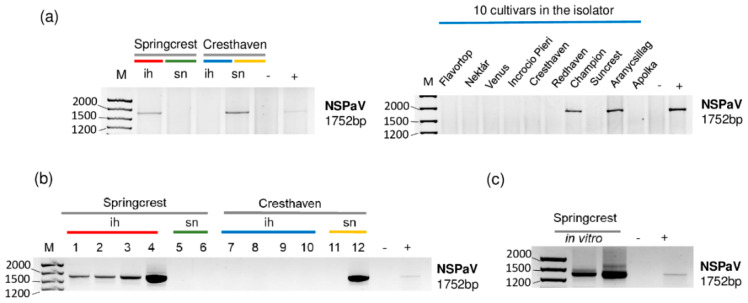
The result of the RT-PCR validation for the presence of NSPaV in the sRNA HTS libraries and individual trees (**a**,**b**) using NSPaV-specific primers amplifying a 1752 bp virus-specific amplicon. (**c**) The result of the RT-PCR validation of NSPaV in in vitro plantlets whose ancestors were used using NSPaV-specific primers amplifying a 1752 bp virus-specific amplicon. M is a GeneRuler 100 bp from Thermo Scientific (Waltham, MA, USA). ih: isolator house; sn: stock nursery; + is a positive control; − is a negative control.

The NSPaV-derived sRNAs were mostly 21–22 nt long, suggesting their DICER (DCL) 2/4 origin. The presence of NSPaV was validated by RT-PCR (Figure 4).

The investigation of each individual tree sampled for sRNA HTS for the presence of NSPaV using RT-PCR revealed that all of the Springcrest trees in the isolator, one out of two Cresthaven trees at the stock nursery and two trees, Champion and Aranycsillag, in the isolator were infected (Figure 4a,b).

In vitro Springcrest plantlets were also infected with NSPaV, which could explain the source of infection in the case of this cultivar (Figure 4c). However, the real source of infection is not that obvious, because Springcrest, Champion and Aranycsillag in the isolator house, and one Cresthaven tree in the open field, were infected. This means that infection both in the isolator and in the open field could occur. NSPaV was previously reported in a symptomatic orchard in Hungary [10], but apart from this description, as far as we know, its presence has not been directly shown in Europe.

**Figure 5:**
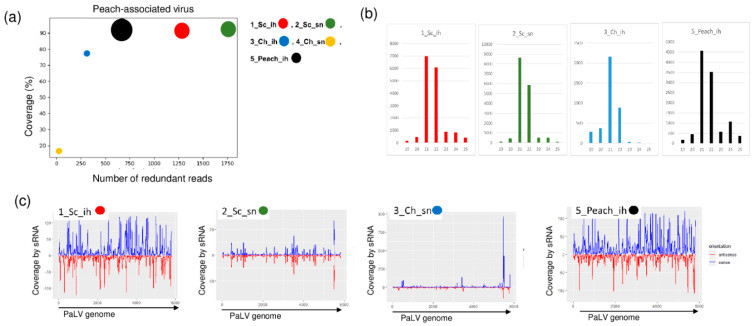
The result of the sRNA HTS and its validation for PaLV in the five sRNA libraries. (**a**) The graphic representation of the result of the bioinformatics analysis of sRNA HTS. The coverage of the PaLV genome (*y*-axis) together with the number of RPM (number of PaLV-specific non-redundant reads in 1 million sequenced reads on the *x*-axis) was plotted as a circle whose diameter is proportional to the number of contigs. Different colors were used for the five libraries, as indicated. (**b**) The size class distribution of the PaLV-derived reads in the 1_Sc_ih, 2_Sc_sn, 3_Ch_ih and 5_Peach_ih libraries (**c**) PaLV-derived reads in the 1_Sc_ih, 2_Sc_sn, 3_Ch_ih and 5_Peach_ih libraries are shown as they mapped to the PaLV genome, indicating their sense and antisense origin (Sc: Springcrest; Ch: Cresthaven; ih: isolator house; sn: stock nursery).

**Figure 6:**
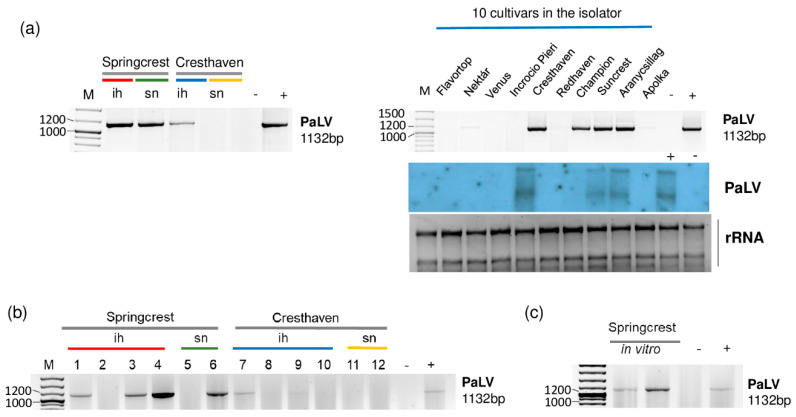
The result of the validation for the presences of PaLV in the sRNA HTS libraries (**a**) and individual trees (**b**) by RT-PCR using PaLV-specific primers amplifying 1132 bp virus- specific amplicon and by Northern blot using a PaLV-specific radiolabeled probe. (**c**) The result of the RT-PCR validation of NSPaV in in vitro plantlets whose ancestors were used using PaLV-specific primers amplifying a 1132 bp virus-specific amplicon. M is a GeneRuler 100 bp from Thermo Scientific (Waltham, MA, USA). ih: isolator house; sn: stock nursery; + is a positive control; − is a negative control.

We found PaLV-specific contigs in 1_Sc_ih, 2_Sc_sn and 5_peach_ih (Table 2). The coverage of the genome was higher than 90% in these three libraries. Moreover, PaLV coverage by sequenced reads was 76.9% in 3_Ch_ih. The 21–22 nt long PaLV-specific reads with both sense and antisense origins covered the entire genome (Figure 5) and the presence of the virus was validated by RT-PCR (Figure 6).

In the 5_Peach_ih library, Cresthaven, Suncrest, Aranycsillag and Champion were infected with PaLV, which was further proven by Northern blot hybridization in the first three varieties (Figure 6a). We suspect that the concentration of PaLV RNA was lower in the case of Champion, and this is why we could not detect its presence by Northern blot. We also found ChaLV-specific contigs in 2_Sc_sn and in 5_Peach_ih (Table 2). When we reannotated these two contigs, we found that they were 100% identical to both ChaLV and PaLV. ChaLV is a *Prunus*-infecting luteovirus described from the Czech Republic [29], which has 73% identity to PaLV. ChaLV-specific contigs in the two libraries were short, only 37 nt long. In this case, they could have been false positives due to the blast annotation. Three Springcrest trees (out of four), one Cresthaven tree (out of four) in the isolator and one Springcrest tree of the two in the stock nursery were infected with PaLV (Figure 6b). Because of this uneven infection, it cannot be suggested that the sources of the infection were the in vitro lines; however, both of the Springcrest in vitro lines were infected with PaLV (Figure 6c).

### 2.4. Frequent Presence of PLMVd in the Tested Trees Was Shown by sRNA HTS and RT-LAMP, but Not with Biological Assays on GF305

**Figure 7:**
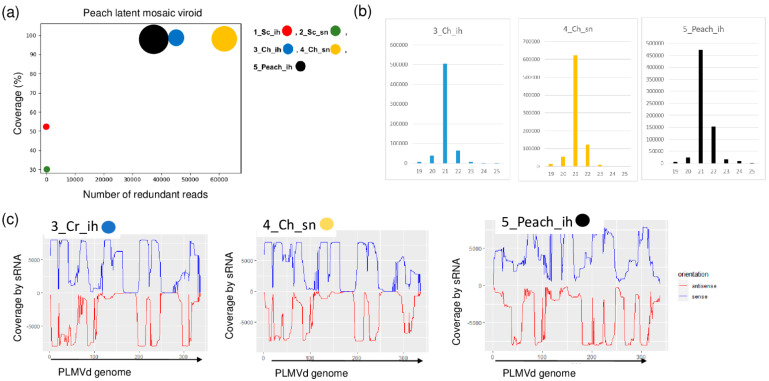
The result of the sRNA HTS and its validation for PLMVd in the five sRNA libraries. (**a**) The graphic representation of the result of the bioinformatics analysis of sRNA HTS. The coverage of the PLMVd genome (*y*-axis) together with the number of RPM (number of PLMVd-specific non-redundant reads in 1 million sequenced reads on the *x*-axis) was plotted as a circle whose diameter is proportional to the number of contigs. Different colors were used for the five libraries, as indicated. (**b**) The size class distribution of the PLMVd-derived reads in 3_Ch_ih, 4_Ch_sn and 5_Peach_ih libraries (**c**) PLMVd-derived reads in 3_Ch_ih, 4_Ch_sn and 5_Peach_ih libraries are shown as they mapped to the PLMVd genome, indicating their sense and antisense origin (Sc: Springcrest; Ch: Cresthaven; ih: isolator house; sn: stock nursery).

PLMVd-specific contigs were present in three of the sRNA libraries: 3_Ch_ih, 4_Ch_sn and 5_Peach_ih. The RPM was very high (43, 63 and 38 thousand in the three libraries, respectively) and the coverage of the viroid genome by the mostly 21 nt long viroid-specific reads was 100% (Figure 7a,b).

**Figure 8:**
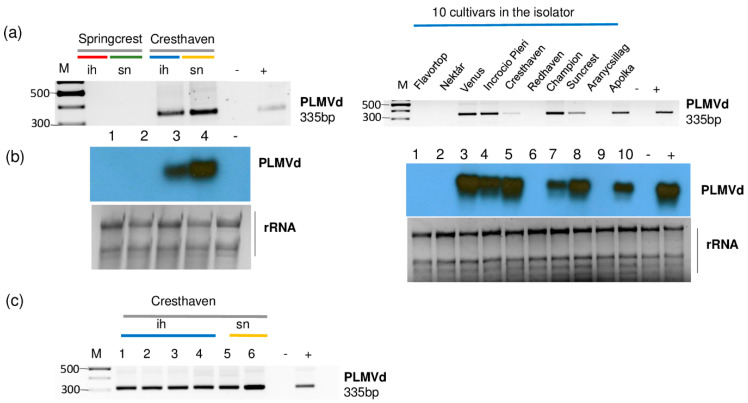
The result of the validation for the presences of PLMVd by (**a**) RT-PCR using PLMVd-specific primers amplifying a 335 bp viroid-specific amplicon or (**b**) Northern blot using a PLMVd-specific radiolabeled probe in the sRNA HTS libraries. (**c**) The result of the RT-PCR validation for the presences of PLMVd in individual Cresthaven trees using viroid-specific primers amplifying a 335 bp viroid-specific amplicon. M is a GeneRuler 100 bp from Thermo Scientific. ih: isolator house; sn: stock nursery; + is a positive control; − is a negative control.

The presence of PLMVd was validated by both RT-PCR and Northern blot hybridization (Figure 8).

All of the sampled Cresthaven trees (both in the isolator house and in the open field) and six trees out of ten in 5_Peach_ih (Venus, Incrocio Pieri, Cresthaven, Champion, Suncrest and Apolka) were infected (Figure 8b,c). Unfortunately, we did not have the in vitro cultured Cresthaven lines which served for the propagation of this cultivar. This is why we could not test if they were the original source of infection.

The cultivars of the 5_Peach_ih were tested for the presence of PLMVd by a biological assay using a GF305 indicator. The survival rate of the grafts was very high and enabled a trustworthy evaluation (Table S3) in the third year. According to this evaluation, no PLMVd infection was detected in all of the investigated trees. However, molecular investigation by Northern blot gave a strikingly different result. The indicators (a pool of all of the trees according to the cultivars) testing PLMVd-infected trees (i.e., Venus, Incrocio Pieri, Cresthaven, Champion, Suncrest and Apolka) contained PLMVd (Figure S1a). Moreover, all of the indicators for positive cultivars were infected, showing that the graft transmission was always successful, although it never corresponded to the presence of symptoms (Figure S1b).

To test an alternative assay for PLMVd, we tried colorimetric RT-LAMP in the case of cultivars of 5_Peach_ih. The result of the assay coincided with the RT-PCR either purified RNA or crude extract was used as a template (Figure S2), indicating PLMVd infection in Venus, Incrocio Pieri, Cresthaven, Champion, Suncrest and Apolka.

### 2.5. Infection with Luteoviruses and PLMVd Are Frequent in the Isolator, but Cannot Be Ascribed as the Only Cause of the Trees’ Decline

**Figure 9:**
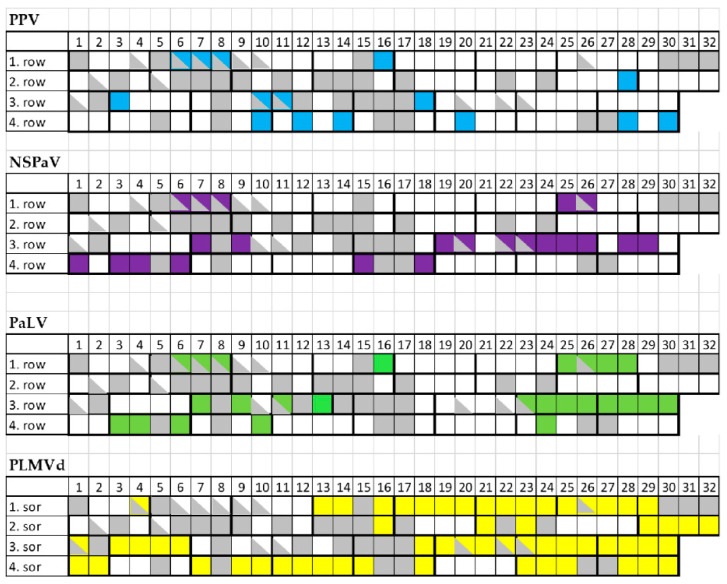
A summary of the virus diagnostic surveys investigating all the individual trees in the peach isolator house. Virus-infected trees are shown by different colors: PPV: blue; NSPaV: violet; PaLV: green; PLMVd: yellow.

To find out if the decline in the isolator can be connected to the infection of the viruses or PLMVd detected by HTS, we surveyed all of the surviving individuals. For the survey we used RT-PCR and Northern blot to detect viruses and PLMVd, respectively (Figure S3). A total of 65 out of the 89 tested trees were virus or viroid infected. A total of 15 trees tested positive for PPV, 22 for NSPaV, 25 for PaLV and 59 for PLMVd (Figure 9).

### 2.6. Phylogenetic Analysis of the Viral Strains

**Figure 10:**
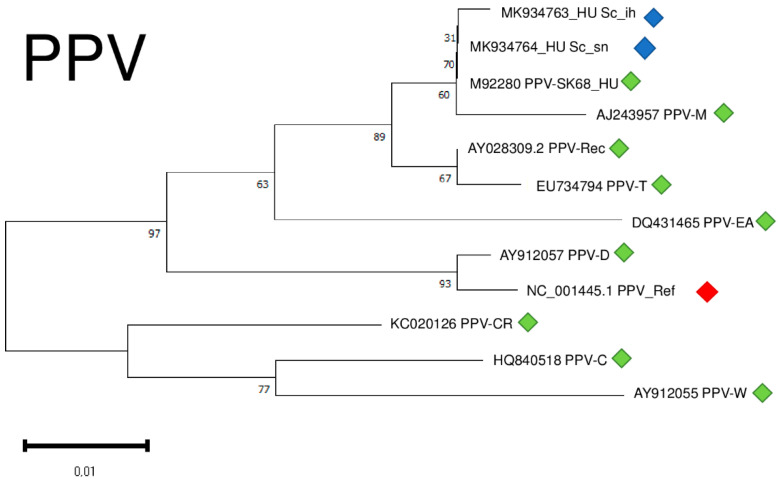
Evolutionary analysis of 427 bp, partial CP coding regions of different PPV isolates by Maximum Likelihood method. The evolutionary history was inferred by using the Maximum Likelihood method and Tamura-Nei model. The tree with the highest log likelihood (−921.09) is shown. The percentage of trees in which the associated taxa clustered together is shown below the branches. Initial tree(s) for the heuristic search were obtained automatically by applying Neighbour-Join and BioNJ algorithms to a matrix of pairwise distances estimated using the Tamura-Nei model, and then selecting the topology with superior log likelihood value after 1000 Bootstraps. Alignment of the sequences was done using MUSCLE. Evolutionary analyses were conducted in MEGA11. Red box is the reference genome, green boxes indicate PPV strains from the NCBI GenBank, while blue boxes indicate PPV isolates from the Hungarian isolator houses.

Although we amplified only a part of the viral coat protein coding region of PPV, we conducted a phylogenetical analysis of the two different isolates. Our isolates clustered together with a PPV_SK68 isolate sequenced in Hungary [30] and with a typical PPV-M isolate shown to preferably occur in peach [31] (Figure 10).

Isolates were 95.8–96.1% identical to the reference genome at the nucleotide level, generating only one amino acid change in the encoded coat protein (CP) (Table S4).

To find out the phylogenetic relationship within the different strains of other viruses, we cloned and sequenced several variants of NSPaV, PaLV and PLMVd (Table S4).

**Figure 11:**
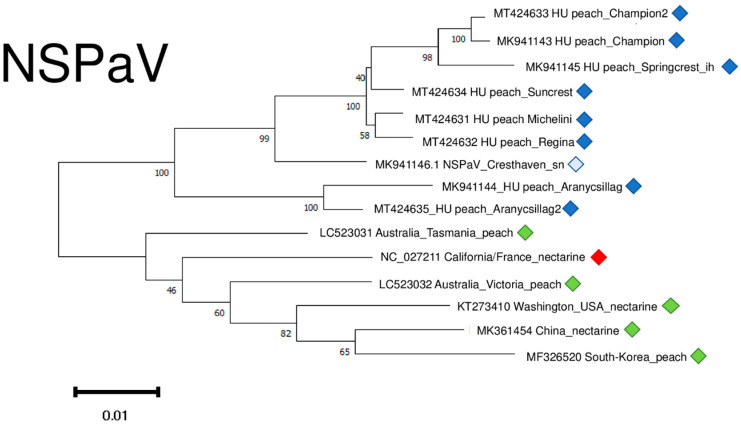
Evolutionary analysis of 1752 bp long part of the NSPaV genome encoding partial CP and readthrough protein of different NSPaV isolates by Maximum Likelihood method. The evolutionary history was inferred by using the Maximum Likelihood method and Tamura-Nei model. The tree with the highest log likelihood (−5007.67) is shown. The percentage of trees in which the associated taxa clustered together is shown below the branches. Initial tree(s) for the heuristic search were obtained automatically by applying Neighbour-Join and BioNJ algorithms to a matrix of pairwise distances estimated using the Tamura-Nei model, and then selecting the topology with superior log likelihood value after 1000 Bootstraps. Alignment of the sequences was done using MUSCLE. Evolutionary analyses were conducted in MEGA11. Red box is the reference genome, green boxes indicate NSPaV strains from the NCBI GenBank, Hungarian NSPaV strains are indicated with dark blue (originating from the isolator house), light blue (originating from the open field stock nursery).

NSPaV variants clustered into two distinct clusters, apart from any other NSPaV found in the NCBI GenBank (Figure 11). It suggests a common “in country” or “in place” origin. The sequence of the variants which were determined in the symptomatic orchard in Hungary clustered distantly from these two clades, together with other variants of different geographical origin. (Figure S4).

NSPaV variants were 93–94.8% identical to the reference genome on the nucleotide and 93–94.1% identical on the amino-acid level (Table S4).

**Figure 12:**
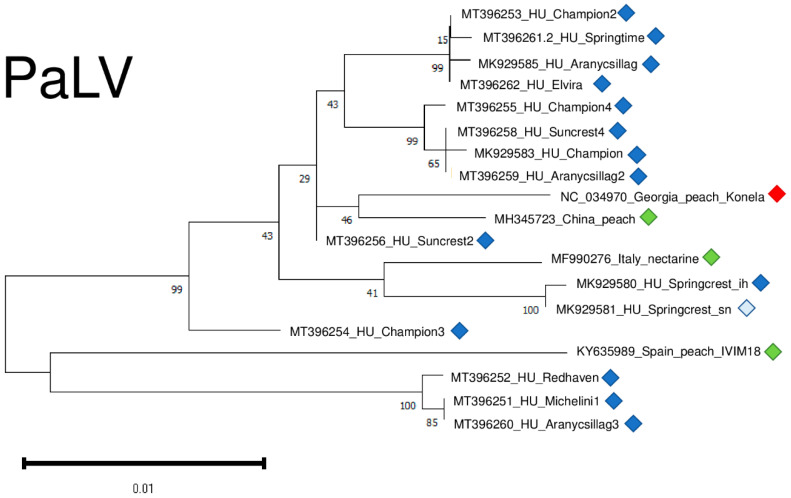
Evolutionary analysis of 1132 bp long part of the PaLV genome encoding partial ORF3a, CP and MP of different PaLV isolates by Maximum Likelihood method. The evolutionary history was inferred by using the Maximum Likelihood method and Tamura-Nei model. The tree with the highest log likelihood (−2214.35) is shown. The percentage of trees in which the associated taxa clustered together is shown below the branches. Initial tree(s) for the heuristic search were obtained automatically by applying Neighbour-Join and BioNJ algorithms to a matrix of pairwise distances estimated using the Tamura-Nei model, and then selecting the topology with superior log likelihood value after 1000 Bootstraps. The tree is drawn to scale, with branch lengths measured in the number of substitutions per site. Alignment of the sequences was done using MUSCLE. Evolutionary analyses were conducted in MEGA11. The red box is the reference genome, green boxes indicate PaLV strains from the NCBI GenBank, Hungarian PaLV strains are indicated with dark blue (originating from the isolator house) and light blue (originating from the open field stock nursery).

Only in recent years has it been described why there are not too many different variants for the reliable phylogenetic analysis of PaLV. However, the tree showing PaLV’s phylogenetic relationships shows that PaLVs from nectarine and peach cluster independently and do not cluster according to their geographical origin (Figure 12).

PaLV variants were very conserved, being 98.1–99% identical to the reference genome on the nucleotide and 95.1–98.6% identical on the amino-acid level (Table S4).

We sequenced variants from different individuals from the same cultivars (Champion, Suncrest, Cresthaven, and Aranycsillag). They did not cluster together, suggesting their independent origin.

**Figure 13:**
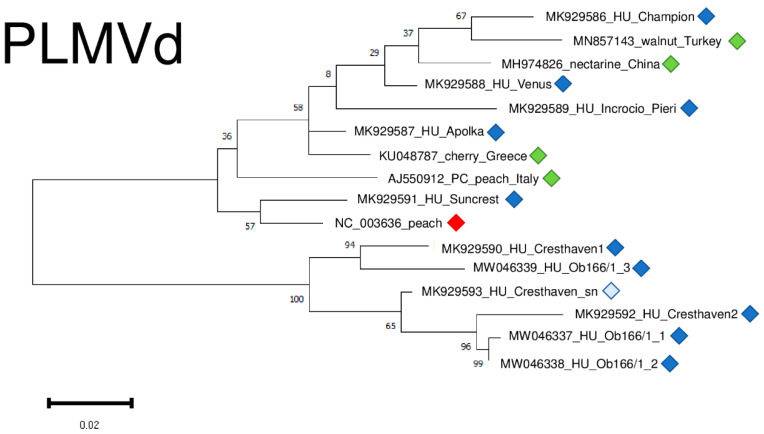
Evolutionary analysis of PLMVd variants, investigating whole genome sequences of different PLMVd isolates by Maximum Likelihood method. The evolutionary history was inferred by using the Maximum Likelihood method and Tamura-Nei model. The tree with the highest log likelihood (−1400.57) is shown. The percentage of trees in which the associated taxa clustered together is shown below the branches. Initial tree(s) for the heuristic search were obtained automatically by applying Neighbour-Join and BioNJ algorithms to a matrix of pairwise distances estimated using the Tamura-Nei model, and then selecting the topology with superior log likelihood value after 1000 Bootstraps. The tree is drawn to scale, with branch lengths measured in the number of substitutions per site. Alignment of the sequences was done using MUSCLE. Evolutionary analyses were conducted in MEGA11. Red box is the reference genome, green boxes indicate PLMVd strains from the NCBI GenBank, Hungarian PLMVd strains are indicated with dark blue (originating from the isolator house), light blue (originating from the open field stock nursery).

The result of our virus diagnostic survey showed that 73% of the individuals were infected with PLMVd. The viroid possesses high genetic variability and we found several point mutations (Figure S5), but the structure of the viroid was not affected by these SNPs. PLMVd variants were very diverse, showing 85.8–95.6% identity to the reference genome (Table S4) and clustered into two clades (Figure 13). It is interesting to remark that different variants originating from two trees, Cresthaven and Ob166, clustered together, showing their same origin and suggesting that they evolved in the tested tree (Figure 13).

**Figure 14:**
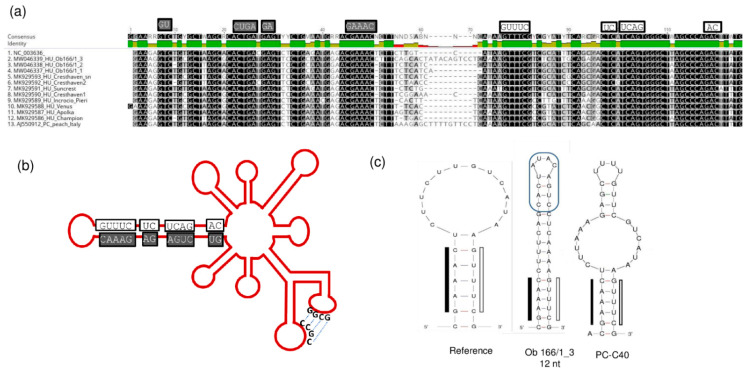
The conserved motifs in the hammerhead structure are highlighted in the (**a**) alignment of the hammerhead arm section of the PLMVd genome of the PLMVd variants and (**b**) on the schematic representation of the viroid genome. (**c**) The altered sequences and structure of the edge of the hammerhead arm in the reference genome, in the PC-C40 variant and in Ob166/1_3. (**b**) was designed according to [13].

The conserved regions in the hammerhead arm of the viroid responsible for its replication are all maintained, together with the “kissing-loop” side chains (Figure 14).

Furthermore, in one case (Ob_166 tree), we identified a PLMVd variant with a 12 nt long insertion located at the same position where the pathogenic determinant of the peach calico (PC) phenotype was found [15] (Figure 14c). This insertion did not show any sequence homology to the PC variant, which is why it is not surprising that its presence did not coincide with the albino phenotype. From this tree we have sequenced three PLMVd variants, but the other two did not have this insertion. The presence of multiple variants from the same viroid in a tree suggests that PLMVd can quickly evolve within its host. Different variants can survive if their sequence is not affected so much that they lose their functionally important secondary structure.

## 3. Discussion

Certified mother trees used as a source of propagation material have to be free from any damaging pathogens. Traditionally, the virus-free status of these trees is tested by biological assays using virus-sensitive indicators to detect any possible infection. These tests are land and labor-consuming, requiring up to three years to obtain a result. Serological methods (ELISA) and molecular PCR-based methods can only test the presence of pathogens whose presence is anticipated in the sample. They are valid alternatives to biological assays to test regulated viruses routinely. The concentration of the viral titer fluctuates during the season, which can directly affect the result of these tests, whose sensitivity is also variable. New variants, bearing mutations within the primer sequence or in the antibody-recognized part of the genome, can escape from the detection. While lateral strip-based ELISA can be used in the field, PCR-based methods need a laboratory and experienced technicians to proceed. In recent years, isothermal amplification methods (LAMP and recombinase polymerase amplification (RPA)) [32] emerged. These tests do not need temperature cycling, the involved DNA polymerases are less sensitive to inhibitors, and the tests can be carried out from crude extract. Thus, they represent easy-to-use, field-compatible alternatives. HTS offers a unique, fast and sensitive alternative and is able to detect all of the presenting viruses in certified plantations. Peach and nectarine have been previously analyzed by HTS in several studies leading to the description of viromes of different peach cultivars [22], symptomatic nectarine trees [21] and the discovery of new peach-infecting viruses [7]. During our work, we surveyed peach cultivars in a germplasm collection using small RNA HTS.

The bioinformatics analysis of the sequenced reads was applied according to our in-house pipeline based on CLC Genomic Workbench. The reliability of bioinformatics pipelines during small RNA HTS analysis is very different [33] and we tried to end up with a representation of different parameters which could be easily used for successful diagnostics. Our pipeline includes BLAST-ing contigs prepared from small RNA reads and the direct mapping of the sequenced reads to the genomes of viruses and viroids that have reference genomes in the NCBI GenBank. For trustworthy results, we set the following threshold limits, based on our previous results: (i) the presence of a virus-specific contig, (ii) an RPM higher than 200 and (iii) the coverage of the viral genome above 60%. The graphic representation of these parameters on a diagram helped us to create a list of viruses, the presence of which could be validated later. If the dot representation of the library was above that of the diagonal on this diagram, the presence of the virus or viroid could be validated.

The size distribution and coverage of the viral genome by virus-specific reads are good indicators of the virus’ presence. Viral specific reads are usually 21–22 nt long, as they are products of the host DCL4 and DCL2 enzymes, respectively, and they usually cover most of the viral genomes by sRNAs that have both sense and antisense orientations [6]. However, we cannot explain the high number of 24 nt long PPV-specific reads in 2_Sc_sn. For the viruses whose presence could be validated, we found these characteristics. Our results show that small RNA HTS is a valid diagnostic method for peach.

sRNA HTS proved its usefulness, and its cost is constantly decreasing. Moreover, we can prepare mixtures, i.e., pools of different individuals before library preparation, and validate the presence of viruses in all of these individuals later on. In the case of 5_Peach_ih, we followed this strategy. We were able to validate the presence of a virus, NSPaV, even if it was present in two out of ten samples, whose RNA was used in the mixture.

The validation of sRNA HTS was performed by different, unrelated methods: RT-PCR, Northern blotting, RT-LAMP and a biological assay. For the first three methods, sequence information about the investigated pathogen was essential to be able to design primers for their amplification and for probe synthesis. Primers used for the validation of the strains present in this research were checked before application by comparison with the consensus sequence of the specific virus prepared from the sequenced small RNA reads. We used previously described primers in the case of PLMVd, but we kept one published primer for PPV diagnostics, fulfilling this requirement. As viruses and viroids evolve fast, this strategy is absolutely essential for correct diagnosis.

In contrast to these sequence-specific validation methods, a biological assay can detect any pathogen in the tested cultivar but only if it induces symptoms on a sensitive indicator. Although GF305 is believed to be sensitive to PLMVd infection, we failed to detect it in any of the investigated samples, even if the viroid was present in the grafts. The biological assay lasted for years and because of the latency of the viroid, it failed to diagnose it.

Our results on this small pilot test show that there is an urgent need to revise the ability of the biological assay as a current official virus diagnostics method in order to prevent the unwanted dissemination of viruses by not precisely diagnosing the propagation material.

RT-PCR and Northern blot are molecular methods which require practice and special hardware, which is why applying them in fast routine assays is difficult. In contrast, RT-LAMP offers a fast, reliable and sensitive alternative. Our results show that RT-LAMP could detect all of the PLMVd-infected cultivars, even if crude extract was used as a template. We used the colorimetric version of RT-LAMP, the result of which can be easily evaluated. This isothermal test works at 65 °C and does not need any special equipment, which is why it could even be carried out in the field or orchard.

SRNA HTS made it possible for us to diagnose the frequent infection of NSPaV and PLMVd and also the presence of PaLV, a virus which had not been previously described in Hungary. Surveying every single individual in an isolator house showing decline revealed a high rate of infection even in these controlled circumstances. The investigation of the location of the trees and the phylogenetic analysis of the presenting variants did not give us any clues for finding the original sources of infection. However, as we found PPV, NSPaV and PaLV in the in vitro culture of Springcrest, it is highly possible that the original sanitized plants were the source of infection.

Shoot tip grafting offers a very promising possibility for virus elimination from peach [34], but this technique has not been widely spread and it is not used in Hungarian routine sanitation protocols. NSPaV and PaLV have been described only in recent years and their presence has not been known in the country. Therefore, it is not surprising that these certified trees were not tested for their presence. PLMVd infection was tested regularly by a biological assay, but we showed that this method is unable to successfully diagnose its presence. The presence of PPV was also very surprising, but the regular tests for PPV were conducted by ELISA in the summer. At that time, high temperatures in the isolator provide good conditions for antiviral silencing. When RNAi against viruses works efficiently, the concentration of virus-derived siRNAs can be high, but the concentration of the intact virus RNA and viral proteins is very low, which could lead to false negative serological diagnostics.

We found mixed infections on several occasions. During our surveys, the decline in additional trees occurred. Although in some cases it coincided with the presence of mixed viral infections, there were cases where no virus or PLMVd were detected in the declined tree while their causative agent could not be identified. The distribution of virus-infected trees did not follow any pattern, and due to their high frequency, it is very difficult to identify the origin of their infection. We showed that the PPV infection of the Springcrest cultivar could originate from its in vitro source.

We could not correlate the presence of viruses to any specific symptoms and not even to the decline in trees. This does not mean that infection did not play any role in the decline, but it is also possible that other factors, i.e., non-viral pathogens or abiotic effects alter or expedite their synergistic effect.

Connecting the presence of the pathogen to any specific symptoms requires the molecular characterization of the viruses described by HTS, which needs special attention in the future [7,9].

## 4. Materials and Methods

### 4.1. Plant Material and Sample Preparation

During our survey in 2017, we sampled individuals of two peach cultivars (Springcrest and Cresthaven) both in the isolator and in open field stock nursery together with representative members of ten cultivars kept in the isolator (Figure 1 and Table 1). The isolator house used for the peach trees was covered by an aphid proof net, which was able to accommodate 128 trees (32 trees in four lines). Leaf samples from four different branches of the trees were collected from the isolator house and open field stock nursery in Central Hungary, Érd, Elvira major. RNA was extracted combining materials of four leaves originating from one tree using the CTAB method [35]. RNA pools for further experiments were prepared by mixing equal amounts of RNA originating from different individuals of the same cultivar (in the case of library 1–4) or from different cultivars (in the case of library 5) as detailed in Table 1. These pools were used for sRNA library preparation (five libraries in total) using the TruSeq Small RNA Library Preparation Kit (Illumina, San Diego, CA, USA) and our modified protocol [36] and were sequenced using a single index on a HiScan2000 by UD GenoMed (Debrecen, Hungary) 50 bp long and single end reading. Fastq files of the sequenced libraries were deposited to the NCBI GEO database and can be accessed through the series accession number GSE130859.

### 4.2. Pipeline for the Data Evaluation of the HTS Results (Bioinformatics)

For bioinformatics analysis, we used CLC Genomic Workbench. After trimming and quality control, longer contigs were built de novo from the non-redundant reads by employing an assembler of CLC (de novo assembly) using the default options: word size 20, bubble size 50, and simple contig sequences with a minimum 35 nt length (see Table S1 for the initial statistics of the analysis). To diagnose the presence of known viruses, we followed two strategies and used Qiagen CLC Genomic Workbench: (i) we built longer contigs from the non-redundant reads and BLAST-ed the result in contigs to the reference genomes of plant hosted viruses downloaded from GenBank. In parallel, (ii) we directly mapped contigs to the reference genome of *Prunus*-infecting viruses. The annotation of these contigs was performed using the BLASTN algorithm with the default options (thread 1, word size 11, match 2, mismatch 3, gap cost existence 5, and extension 2) and the NCBI plant-hosted viral reference genomes (downloaded at 30 June 2021). For the viruses detected by this method (i.e., when at least one virus-specific contig was present), the reads were directly mapped to the reference genome and were counted with and without redundancy (using the map to the reference command allowing 1 mismatch). The number of normalized reads (read/1 million reads: RPM) was then calculated from the mapped redundant reads and the number of total sequenced reads. The coverage (%) of the viral genome was calculated based on a consensus sequence generated from this mapping. The presence of the virus was indicated if at least two parameters of the three investigated ones were present: (i) the presence of any virus specific contigs, (ii) a number of normalized redundant virus specific reads >200, or (iii) the coverage of the virus genome was >60% or the coverage of the viroid genome was >80% fulfilled. The result of this analysis is summarized in Table 2. To visualize the results of the bioinformatics analysis, we plotted RPM and coverage values for each of the detected viruses using our own script in R Studio. On these plots, the characteristics for the cultivar are marked with a circle, the diameter of which is proportional to the number of virus-specific contigs. We also prepared figures showing the coverage of the genome by sense and antisense virus-specific reads together with the column diagram of the size distribution of virus-derived reads. For the sequence alignment of the viroid variants, we used Geneious Prime 2022.0.2 (https://www.geneious.com).

### 4.3. Confirmation of the Obtained Results by RT-PCR, Northern Blot and Sanger Sequencing

To validate the results of the bioinformatics analysis, RT-PCR and/or Northern blot hybridization with virus-specific primers or virus-specific probes were carried out.

cDNA was synthetized from RNA representing each library or each individual tree using random primer and the RevertAid First Strand cDNA Synthesis Kit (Thermo Fisher Scientific, Waltham, MA, USA), according to the manufacturer’s instructions. The generated cDNA was used as templates for PCR reactions using Phire Hot Start II DNA Polymerase (Thermo Fisher Scientific, Waltham, MA, USA) and published diagnostic primers or new ones (Table S2), which were designed according to the sequenced sRNA reads. PCR products were analyzed by agarose gel electrophoresis. For Sanger sequencing, cDNA was synthetized from the RNA extract of individual plants and virus-specific PCR was performed using Q5 High-Fidelity DNA Polymerase (New England Biolabs, Ipswich, UK). The purified products were cloned into pJET vector System (Thermo Fisher Scientific, Waltham, MA, USA) and sequenced. Sequences were deposited into GenBank (GenBank accession numbers: MK929579-93, MK934763-4, MK941143-6, MT396251-MT396262, MT424631- MT424635, and see Table S4).

For Northern blot analyses, 1–3 µg of total RNA was separated on formaldehyde-1.5% agarose gel and blotted to the Amersham Hybond-NX membrane (GE Healthcare and Life Sciences), by the capillary method using 20x SSC (0.3M NaCl, 30 mM Na-citrate (pH 7.2)). Hybridization was carried out at 65 °C in Church buffer (0.5M sodium phosphate buffer, pH 7.2 containing 1% BSA, 1 mM EDTA, 7% SDS) overnight with the appropriate radioactively labelled probe, washed for 5 min in 2× SSC, 0.1% SDS and for 15 min in 0.5× SSC, 0.1% SDS at the temperature of the hybridization and exposed to an X-ray film. Virus-specific, P32-labelled DNA probes were prepared by using the DecaLabel DNA Labeling Kit (Thermo Fischer Scientific, Waltham, MA, USA). As a template for radioactive probe preparation, the PCR-amplified and purified PCR product of the cloned region of the viruses were used.

### 4.4. RT—LAMP Assay for PLMVd Detection

The RT-LAMP test was performed using the WarmStart Colorimetric LAMP kit from New England Biolabs (Ipswich, UK). Previously published primers were diluted by 10 mM Tris pH 8 [16] (see Table S2 for their sequences). In the reaction mixture, the final concentrations of the primers were 0.2 µM (F3 and B3) and 2.0 µM (FIP and BIP). For crude extract preparation, we used the solution of the OptiGene LAMP kit (OptiGene). A total of 200 mg of frozen leaves was grinded in a mortar in lysis buffer of (OptiGene). A total of 10 µL of this crude sap was added to 50 µL dilution buffer (OptiGene). The reaction mixture, including 2 µL of purified RNA or crude extract, was incubated at an isothermal temperature of 65 °C for 30 min and was then cooled to room temperature before being photographed.

### 4.5. Biological Assay

Buds of the certified trees were graft transmitted to *Prunus avium*, *Prunus persica* GF 305, *Prunus* hibrid GF 31 and *Prunus serrulata* Kwanzan in August 2016. The sprouting rate was determined after a year (Table S3). The symptoms on the indicators were evaluated in the first, second and third year. Leaf samples from the Prunus persica GF 305 indicator were collected in 2019 (in the third year after grafting). From these leaves RNA was isolated and the presence of PLMVd was tested by Northern blot hybridization, as described above.

### 4.6. Phylogenetical Analysis

For phylogenetic analysis we used MEGA11 [37]. Alignment of the sequences was done using MUSCLE embedded in MEGA11. The evolutionary history was inferred by using the Maximum Likelihood method and Tamura-Nei model [38]. The trees with the highest log likelihood were prepared and shown. Initial tree(s) for the heuristic search were obtained automatically by applying Neighbour-Join and BioNJ algorithms to a matrix of pairwise distances estimated using the Tamura-Nei model, and then selecting the topology with superior log likelihood value after 1000 Bootstraps [39]. The trees were drawn to scale, with branch lengths measured in the number of substitutions per site.

## 5. Conclusions

We proved the incidence of PLMVd and PaLV for the first time in Hungary. We suspect that the source of the viral infection might be the propagation material, which was used as a base for the variety collection in this isolator house.

Global markets and climate change affected the habitat and geographical distribution of fruit trees together with their vectors of pathogen diseases, and also insect pollinators [17]. To prevent the total globalization of pathogens and protect both agricultural and endemic plants it is absolutely essential to be aware of the presenting pathogens and all these possible infection sites. HTS is the only method which can reveal all of the presenting pathogens in the sample. Its usage at a quarantine station proved its usefulness and led to the discovery of new viral agents. Based on the results gained by HTS, there is a high demand for new sensible rules and lists of pathogens, the presence of which should be tested at the quarantine stations [8,40,41,42]. For their detection, the use of reliable diagnostic methods should be considered, keeping in mind affordable practices in terms of price, human resources, and time frame, to make their use not only agronomically, but economically beneficial.

## Figures and Tables

**Table 1:** Basic information of the sampled varieties with their respective small RNA library codes. (Sc: Springcrest; Ch: Cresthaven; ih: isolator house; sn: stock nursery).

Library code	Cultivar	Isolator House/Open Field Stock Nursery	Number of Tested Cultivars	Number of Tested Trees
1_Sc_ih	Springcrest	isolator house	1	4
2_Sc_sn	Springcrest	stock nursery/open field	1	2
3_Ch_ih	Cresthaven	isolator house	1	4
4_Ch_sn	Cresthaven	stock nursery/open field	1	2
5_Peach_ih	1_Flavortop	isolator house	10	10
	2_Nektár H			
	3_Venus			
	4_Incrocio Pieri			
	5_Cresthaven			
	6_Redhaven			
	7_Champion			
	8_Suncrest			
	9_Aranycsillag			
	10_Apolka			

**Table 2:** Summary of the bioinformatics analysis. Library codes are the same as those detailed in Table 1 (Sc: Springcrest; Ch: Cresthaven; ih: isolator house; sn: stock nursery).

Library Code	Bioinformatics Analysis	Viruses				Viroid
		PPV	NSPaV	PaLV	ChaLV	PLMVd
**1_Sc_ih**	n of virus specific contigs	94	2	17	0	0
	n of non-redundant reads	3867	1353	2368	677	17
	RPM	4908	462	1296	197	3
	% coverage	68.3	89.7	93.9	48.3	51.6
**2_Sc_sn**	n of virus specific contigs	1	0	13	1	0
	n of non-redundant reads	637	166	2547	714	6
	RPM	122	46	1759	312	1
	% coverage	52.0	32.4	94.3	51.2	291
**3_Ch_ih**	n of virus specific contigs	0	0	0	0	14
	n of non-redundant reads	102	56	1012	244	2817
	RPM	34	9	307	47	43,915
	% coverage	14.2	13.5	76.9	29.4	99.1
**4_Ch_sn**	n of virus specific contigs	0	0	0	0	20
	n of non-redundant reads	143	1291	95	66	3586
	RPM	33	350	16	14	63,289
	% coverage	17.1	81.5	16.5	12.1	100
**5_peach_ih**	n of virus specific contigs	0	16	41	1	89
	n of non-redundant reads	509	1627	3076	848	5462
	RPM	79	239	670	125	38,197
	% coverage	43.9	93.6	98.5	61.1	100

RPM = number of redundant reads/1 million reads. The grey color shows where the set threshold level of the parameters were reached.

## Data Availability

The Fastq files of sRNA HTS are accessible at GEO, series accession number GSE130859. The sequences of the virus variants are available at NCBI GenBank with the following accession numbers: MK929579-93, MK934763-4, MK941143-6, MT396251-MT396262, MT424631.

## Supplementary Materials

The following supporting information can be downloaded at: https://www.mdpi.com/article/10.3390/plants11121591/s1, Figure S1. The result of the validation for the presences of PLMVd by Northern blot; Figure S2. The result of the validation for the presences of PLMVd by colorimetric RT-LAMP assay in the individuals of 5_Peach_ih; Figure S3. The result of the validation for the presences of PLMVd by Northern blot in all of the individual trees in the isolator house; Figure S4. Evolutionary analysis of 411 bp long part of the NSPaV genome encoding partial CP of different NSPaV isolates by Maximum Likelihood method.; Figure S5. The alignment of the sequence of the PLMVd variants, highlighting highly variable region on the PLMVd genome; Table S1: The initial statistics of the small RNA HTS (GEO accession number: GSE130859); Table S2 The sequences of the primers used for virus detection in RT-PCR; Table S3: The sprouting rate efficiency of the indicators evaluated after a year; Table S4: GenBank identifiers of the amplified and sequenced virus specific products.
