# Supplementary material for: Viromes of Hungarian Peach Trees Identified by High-Throughput Sequencing of Small RNAs
Source: Plants (Basel). 2022 Jun 16;11(12):1591. doi: 10.3390/plants11121591 (PMC9230589; doi:10.3390/plants11121591)
Supplement: Supplementary file 1 [file plants-11-01591-s001.zip › plants-1703859-supplementary/Supplementary Figures.pdf]

plants

Supporting S1 to

Viromes of Hungarian peach trees identified by high-throughput sequencing of small RNAs

Daniel Barath, Nikoletta Jaksa-Czotter, Tunde Varga and Eva Varallyay*

* Correspondence: varallyay.eva@uni-mate.hu

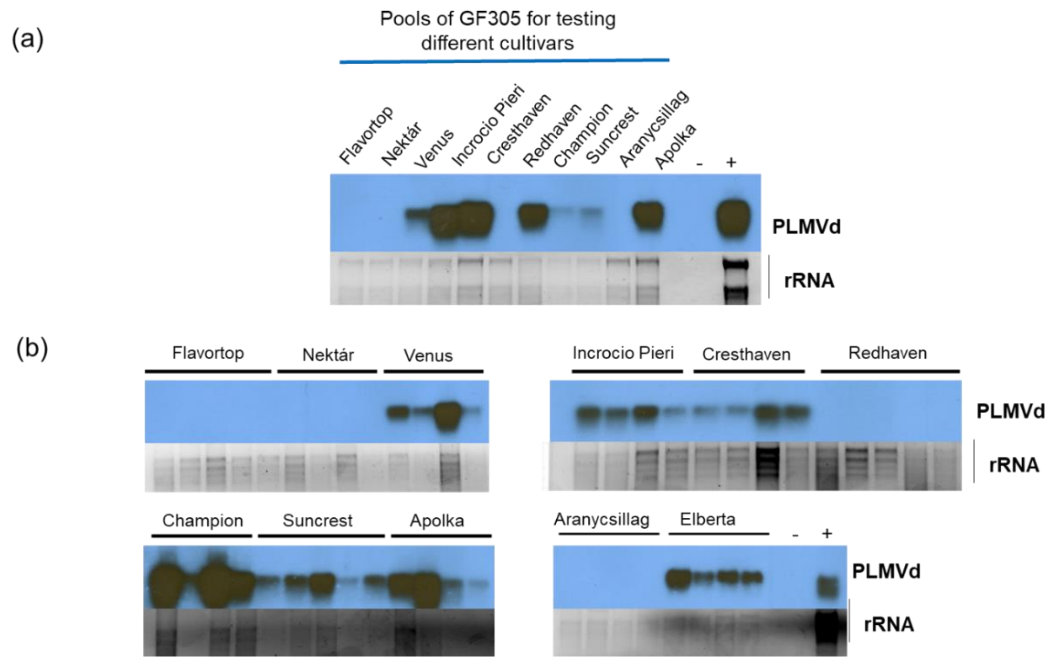

Figure S1. The result of the validation for the presences of PLMVd by Northern blot using PLMVd specific radiolabelled probe in the (a) pool (b) individuals of the GF305 trees grafted by different cultivars. + is a positive control while – is a negative control.

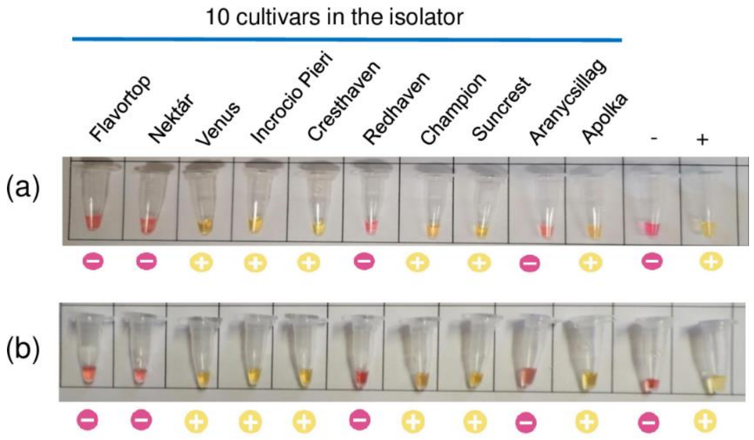

Figure S2. The result of the validation for the presences of PLMVd by colorimetric RT-LAMP assay in the individuals of 5_Peach_ih using (a) purified RNA or (b) crude extract as a template.

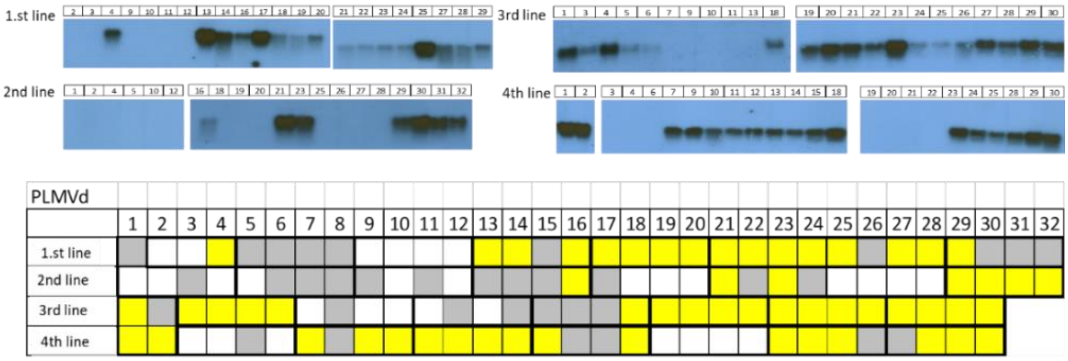

Figure S3. The result of the validation for the presences of PLMVd by Northern blot using PLMVd specific radiolabelled probe in all of the individual trees in the isolator house.

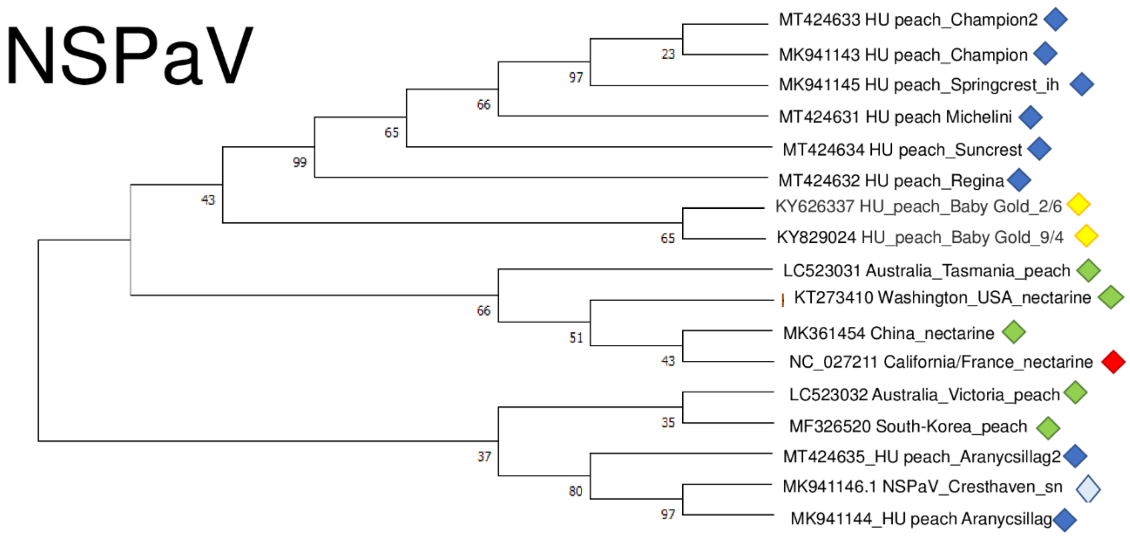

Figure S4. Evolutionary analysis of 411 bp long part of the NSPaV genome encoding partial CP of different NSPaV isolates by Maximum Likelihood method.

The evolutionary history was inferred by using the Maximum Likelihood method and Tamura-Nei model [1]. The tree with the highest log likelihood (-1241.50) is shown. The percentage of trees in which the associated taxa clustered together is shown below the branches. Initial tree(s) for the heuristic search were obtained automatically by applying Neighbor-Join and BioNJ algorithms to a matrix of pairwise distances estimated using the Tamura-Nei model, and then selecting the topology with superior log likelihood value after 1000 Bootstraps [2]. Alignment of the sequences was done using MUSCLE. Evolutionary analyses were conducted in MEGA11 [3].

Red box is the reference genome, green boxes indicate NSPaV strains from the NCBI GenBank, Hungarian NSPaV strains are indicated with dark blue (originating from the isolator house), light blue (originating from the open field stock nursery). Yellow indicates isolates sequenced at a symptomatic Hungarian orchard.

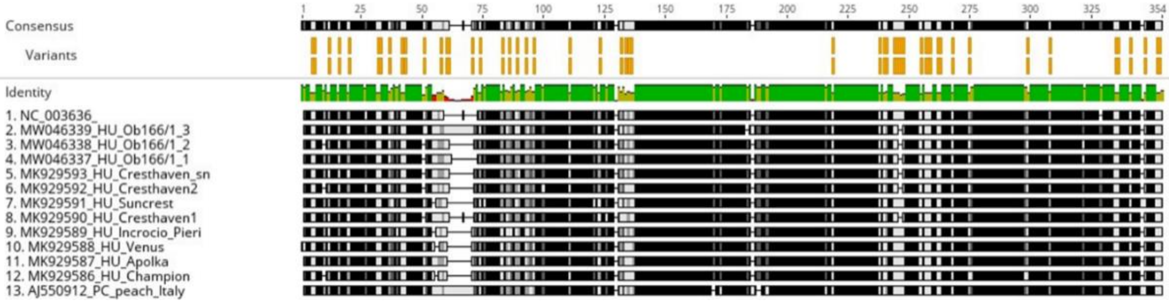

Figure S5. The alignment of the sequence of the PLMVd variants, highlighting highly variable region on the PLMVd genome.

References

1. Tamura, K.; Nei, M. Estimation of the number of nucleotide substitutions in the control region of mitochondrial DNA in humans and chimpanzees. *Molecular Biology and Evolution* **1993**, *10*, 512-526.10.1093/oxfordjournals.molbev.a040023
2. Felsenstein, J. Confidence limits on phylogenies: An approach using the bootstrap. *Evolution; international journal of organic evolution* **1985**, *39*, 783-791.10.1111/j.1558-5646.1985.tb00420.x
3. Tamura, K.; Stecher, G.; Kumar, S. Mega11: Molecular evolutionary genetics analysis version 11. *Molecular Biology and Evolution* **2021**, *38*, 3022-3027.10.1093/molbev/msab120
